# Placental abruption and risk for intraventricular hemorrhage in very low birth weight infants: the United States national inpatient database

**DOI:** 10.1038/s41372-024-02017-y

**Published:** 2024-05-29

**Authors:** Mohsen A. A. Farghaly, Hany F. Aziz, Subhash Puthuraya, Alshimaa Abdalla, Hany Aly, Mohamed A. Mohamed

**Affiliations:** 1 Neonatology Division, Cleveland Clinic Children’s Hospital, Cleveland, OH USA; 2 Faculty of Medicine, Aswan University, Aswan, Egypt

**Keywords:** Risk factors, Brain injuries

## Abstract

**Objective:**

To examine the association of placental abruption with intraventricular hemorrhage (IVH) in very low birth weight (VLBW) infants.

**Methods:**

We examined the National Inpatient Sample (NIS) datasets. Preterm infants <1500 g birth weight (BW) were included. The odds ratios (OR) of developing IVH and severe IVH in association with placental abruption were calculated. Adjusted OR (aOR) were calculated using logistic regression models.

**Results:**

The study included 113,445 VLBW infants. IVH occurred in 18.7% in the infants who were born to mothers with history of placental abruption versus 14.7% in infants without placental abruption, aOR 1.25 (95%CI: 1.13–1.38), *p* < 0.001. Severe IVH occurred in 6.4% in infants born to mothers with history of placental abruption versus 4.0% in those without placental abruption, aOR 1.53 (95%CI: 1.30–1.78), *p* < 0.001.

**Conclusion:**

Placental abruption is associated with increased prevalence of IVH and severe IVH in VLBW infants.

## Introduction

Placental abruption (PA) is the premature separation of the placenta from the uterus before delivery, and it is a serious perinatal condition that is associated with adverse neonatal outcome. Approximately 1% of all pregnancies are complicated by PA, and that rate is potentially higher in preterm deliveries [1–3]. Germinal matrix hemorrhage-intraventricular hemorrhage (GMH-IVH) is a bleeding inside or around the brain’s ventricles, and it is a complication that occurs in preterm infants [4]. It varies in severity; hemorrhage limited to the germinal matrix (grade 1), IVH without ventricular dilatation (grade 2), IVH with ventricular dilatation (grade 3), or intraparenchymal hemorrhage (grade 4) [5]. Severe IVH, grades 3 and 4, are associated with notable morbidity, increased mortality, and significant neurodevelopmental impairment among survivors [6, 7].

It is not known if PA is independently associated with IVH in very low birth weight (VLBW) infants, <1500 g [8]. In preterm infants, autoregulation of cerebral blood flow is limited, thus, the brain is unprotected from fluctuations or rapid changes in blood pressure [9]. Maternal hypotension is a common finding in PA that presents with bleeding and may lead to hypoxic–ischemic injury of the fetal/neonatal germinal matrix [10] that might predispose to IVH. However, the association of PA and IVH has not been adequately studied. Therefore, there is unmet need to study such an association; especially that PA is not uncommon. In this study, we used the United States (US) national database. Our objective was to examine the association of placental abruption with IVH in VLBW infants.

## Methods

This cohort analysis examined a mega database to identify the association of PA in pregnant women with increased risk of IVH and severe IVH in their VLBW infants using the National Inpatient Sample (NIS) in the years 2016–2018.

### Data source

The NIS dataset is produced by the Healthcare Cost and Utilization Project (HCUP) sponsored by the Agency for Healthcare Research and Quality (AHRQ). NIS is a de-identified, publicly available inpatient healthcare database. It contains data of more than 7 million hospital stays each year. NIS uses a stratified, single-stage cluster sampling design, with region, urban/rural location, teaching status, ownership, and bed size to identify strata. After stratification, a random sample of 20% of the hospitals from the target population is included. HCUP program requires the use of weighted samples to reflect national trends. NIS contains information on all patients including patient demographics, primary and secondary diagnoses, primary and secondary procedures, hospital characteristics, payment source, length of stay, and patients’ disposition. All types of admissions were included whether they were direct admissions, admissions from the emergency room or transfers from other hospitals. To avoid duplicate inclusion, infants who were transferred out of the delivery hospital were excluded.

### Patient selection

Preterm VLBW infants were identified in the dataset using the code (NeoMat = 2) that is unique to neonatal hospitalization at birth in addition to the respective International Classification of Diseases codes - 10th version (ICD10) for VLBW infants with respective gestational age (GA) and BW categories. Infants born to mothers with specific medical or perinatal diagnosis were identified using respective ICD-10 codes. Similarly, infants developed postnatal condition or adverse effects were identified using respective ICD-10 codes. For different IVH grades, we used the ICD-10 codes: P520 (grade 1), P521 (grade 2), P522 (grades 3 and 4), P5221 (grade 3), and P5222 (grade 4). For PA, we used the code P021 (we extracted our samples from the neonatal records where all ICD-10 codes are designed for pediatrics population, but the obstetrical codes for PA, O45.9, O45.90, O45.91, O45.92, and O45.93, would be found only under maternal records and could not be used for this study). For all BW categories below 1500 grams, we used the codes: P0715, P0714, P0703, P0702, and P0701. Infants with central nervous system (CNS) anomalies, congenital heart disease (CHD), congenital diaphragmatic hernia (CDH), abdominal wall defects, hypoxic ischemic insult events at birth, and common genetic and chromosomal disorders were excluded from this analysis as any of these diagnoses may act as an independent risk factor associated with adverse neurological outcomes. This study involved publicly available de-identified data; therefore, it was exempted from review by the Institutional Review Board.

### Study design

Infants included in the study were divided into two groups: infants born to mothers who suffered placental abruption and infants born to mothers who did not suffer placental abruption. Demographic, clinical, and perinatal characteristics were compared between the two groups. The odds ratios (OR) to develop IVH or severe IVH in VLBW infants born to pregnant women who suffered placental abruption were calculated using chi square testing. Such association was reexamined using logistic regression models to calculate adjusted OR while controlling for potential confounding variables including maternal conditions (maternal diabetes or hypertension), perinatal occurrences (breech or malpresentation, nuchal cord or cord prolapse, placental previa, or chorioamnionitis), infants’ demographics (sex, GA, BW, multiple gestation, small for gestational age [SGA] status), and postnatal conditions (respiratory distress syndrome [RDS], pneumothorax, pulmonary hemorrhage or hypertension, apnea or anemia of prematurity, necrotizing enterocolitis [NEC], or sepsis).

### Statistical analysis

Binomial and categorical variables were described using frequencies and percentages. Chi-square and Fisher’s exact tests were used to compare groups. Statistical significance was set at *p* < 0.05. Regression analysis was performed to verify significant associations while controlling for confounders. Data analysis was performed using SAS version 9.4 (SAS Institute Inc., Cary, NC).

## Results

The study identified 113,445 VLBW infants in the dataset who fulfilled inclusion criteria. Sample included 49.5% female infants, 35.5% Caucasians, 75.8% singleton pregnancies, 33.0% Cesarean deliveries, and 4.8% small for gestational age infants. Placental abruption occurred in 2.6% among the mothers of these infants. Table 1 demonstrates comparison of the demographic, perinatal and clinical characteristics between the two groups. Infants born to mothers who encountered the adverse event of PA at the time of delivery have also significant association with chorioamnionitis, placenta previa, breech or malpresentation, nuchal cord, higher association of being singleton infants and delivered by Cesarean sections. On the contrary, they have less association with being SGA.

**Table 1:** Demographic, perinatal, and clinical characteristics of in infants born to mothers with placental abruption compared to infants born to mothers without placental abruption.

	Infants born to mothers with placental abruption *n* = 2895	Infants born to mothers without placental abruption *n* = 110,550	OR (95% CI), *P* value
Maternal hypertension	5.7	5.3	1.08 (0.92–1.27), 0.35
Maternal diabetes	4.5	3.7	1.21 (1.01–1.45), 0.04
Maternal chorioamnionitis	4.2	2.9	1.43 (1.19–1.72), <0.001
Placenta previa	1.4	0.4	3.15 (2.28–4.35), <0.001
Breech presentation	6.6	3.5	1.92 (1.65–2.23), <0.001
Malpresentation	1.2	0.4	3.25 (2.30–4.59), <0.001
Nuchal cord	1.6	0.8	1.93 (1.43–2.62), <0.001
Cord prolapse	0.4	0.5	0.75 (0.40–1.40), 0.36
Female sex	49.0	49.6	0.98 (0.91–1.05), 0.53
Race/Ethnicity			
Caucasians	34.9	35.6	Reference
African Americans	29.0	26.5	1.07 (1.02–1.12), 0.82
Hispanic/Latino	16.8	15.5	1.03 (0.98–1.08), 0.11
Asians	3.6	4.3	1.12 (1.03–1.22), 0.22
Native Americans	0.5	0.8	1.05 (0.87–1.27), 0.83
Gestational age ≤28 weeks	62.5	51.7	1.73 (1. 85–1.91), <0.001
Birth weight ≤1000 g	45.4	42.1	0.83 (0.75–0.91), <0.001
Cesarean delivery	75.3	66.7	1.52 (1.39–1.67), <0.001
Small for gestational age	2.6	4.9	0.52 (0.41–0.65), <0.001
Respiratory distress syndrome	67.0	65.3	1.08 (1.00–1.17), 0.055
Pneumothorax	4.0	3.4	1.17 (0.97–1.41), 0.11
Pulmonary hemorrhage	0.2	0.4	0.44 (0.18–1.07), 0.06
Pulmonary hypertension	0.7	1.0	0.71 (0.46–1.11), 0.13
Apnea of prematurity	55.4	52.8	1.11 (1.03–1.20), 0.005
Anemia of prematurity	48.5	45.6	1.12 (1.04–1.21), 0.002
Necrotizing enterocolitis	4.7	5.6	0.83 (0.70–0.99), 0.04
Sepsis	19.3	17.3	1.15 (1.05–1.26), 0.003

All values are percentages.

**Fig. 1:**
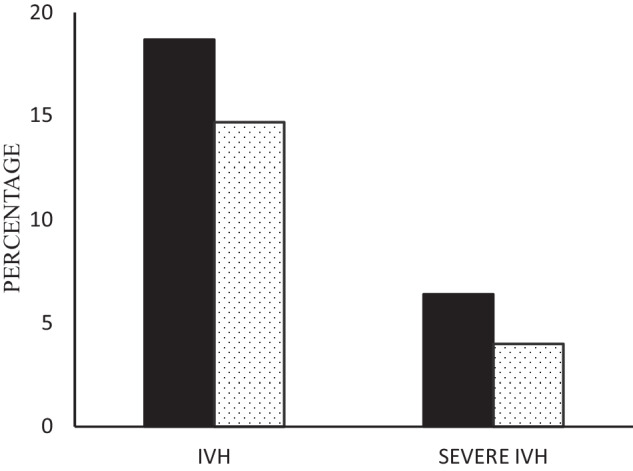
Placental abruption and intraventricular hemorrhages. IVH Intraventricular Hemorrhages (IVH). Black bars represent infants born to mothers with placental abruption (*n* = 2895). White bars represent infants born to mothers without placental abruption (*n* = 110550). For IVH: aOR = 1.25 (95%CI: 1.13–1.38, *P* < 0.001). For severe IVH: aOR = 1.53 (95%CI: 1.30–1.78, *P* < 0.001).

In the overall sample, IVH occurred in 14.8% and severe IVH occurred in 4.1% of the VLBW infants. However, IVH occurred in 18.7% in the infants born to mothers with PA versus 14.7% in infants born to mothers without PA, adjusted OR (aOR) after controlling for the confounding variables above are 1.25 (95%CI: 1.13–1.38), *p* < 0.001. Severe IVH occurred in 6.4% in infants born to mothers with placental abruption versus 4.0% in those without history of placental abruption, adjusted OR 1.53 (95%CI: 1.30–1.78), *p* < 0.001, Fig. 1.

In addition, we examined confounding variables significantly associated with intraventricular hemorrhage in the context of maternal placental abruption, Table 2. Certain factors were associated with increased prevalence of IVH including chorioamnionitis, placenta previa, cord prolapse, nuchal cord, RDS, pneumothorax, pulmonary hemorrhage, pulmonary hypertension, apnea of prematurity, anemia of prematurity, sepsis and NEC. On the contrary, factors such as maternal hypertension, breech or malpresentation, infant sex, singleton status were not associated with increased prevalence of IVH. SGA status was associated with significantly less IVH (OR = 0.52, 95% CI: 0.41–0.65, *P* < 0.001).

**Table 2:** Factors associated with increased prevalence of intraventricular hemorrhage in the study population (*n* = 113,445).

	aOR (95% CI), *P* value of confounding variables for IVH
Placental abruption	1.25 (1.13–1.38), <0.001
Chorioamnionitis	1.43 (1.30–1.57), <0.001
Placenta previa	1.39 (1.07–1.80), =0.01
Cord prolapse	1.59 (1.25–2.04), <0.001
Nuchal cord	1.26 (1.04–1.52), =0.02
Gestational age ≤28 weeks	1.52 (1.45–1.59), <0.001
Birth weight ≤1000 g	1.28 (1.23–1.33), <0.001
Pneumothorax	2.07 (1.90–2.23), <0.001
Pulmonary hemorrhage	3.10 (2.47–3.89), <0.001
Pulmonary hypertension	1.83 (1.58–2.12), <0.001
Apnea of prematurity	1.34 (1.28–1.40), <0.001
Anemia of prematurity	2.71 (2.59–2.84), <0.001
Necrotizing enterocolitis	1.77 (1.65–1.90), <0.001
Sepsis	1.81 (1.73–1.89), <0.001

*aOR* Adjusted Odds Ratio, *IVH* Intraventricular Hemorrhages, *PA* Placental Abruption.

## Discussion

This study demonstrated the association of PA with increased IVH in VLBW infants. In addition, PA was associated with increased prevalence of severe IVH, grades 3 and 4. There are multiple mechanisms that could plausibly explain the increased IVH in PA. Causes of premature placental separation included maternal disseminated intravascular coagulation, sudden mechanical events (e.g., trauma) or rapid uterine decompression [10, 11]. Moreover, decidual bleeding leads to release of tissue factor, thromboplastin, which generates thrombin, that leads to up-regulation of inflammatory cytokines and vascular disruption [12–14]. As well, in chronic abruption, placental inflammation is associated with redistribution of fetal cerebral blood flow [15, 16]. Another possible mechanism may be the underlying cause, maternal cocaine abuse, which induces vasoconstriction leading to ischemia, reflex vasodilatation, and disruption of vascular integrity [17, 18]. PA likely impairs fetal blood flow/utero placental perfusion leading to fetal hypovolemia, hypoxia and acidemia [19]. All these events could be, similarly, risk factors for IVH.

The findings of this study agree with a few small previous reports. PA was associated with a four-fold increased prevalence of periventricular hemorrhage [20]. In forty low birthweight infants (<2500 g) delivered after PA and in 80 control infants of similar gestational age, the prevalence of IVH was 17.5% in the cases and 5% in the controls [21]. Six preterm infants (<927 g) had IVH out of fifteen mothers with PA [22]. On the other hand, a large national study assessed the neonatal outcomes associated with PA but did not look into the IVH [23]. One metanalysis did not show a difference in the odds of IVH in infants born to women with PA. However, according to the metanalysis’s authors, they included only eight studies with overall poor quality and high heterogeneity, sample size was small, did not have sufficient power to detect statistical differences, and they were unable to examine confounders [24]. The IVH to which PA would be directly related is the IVH that occur in the immediate postnatal period. Although epidemiologic studies and ICD codes can not reassure the contemporaries of the events. However, it is confirmed in previous studies that 90% IVH occur in the first 3 days of life, and almost all of them occurs by first week of life [25]. This study showed that SGA status was associated with significantly less IVH. This finding agrees with a previous study that reported a significantly decreased rate of IVH in the SGA [26]. Another surprising finding is that NEC was lower in the PA group, but we could not find a plausible explanation for that.

This study has several strengths. To our knowledge, it is the largest to assess the relationship between PA and IVH. The study sample is representative to all deliveries in the U.S. providing a precise estimate for the prevalence of IVH in more than hundred thousand of newborns with VLBW. The robust number of sociodemographic and clinical covariates have allowed to control for potential confounders. We recognize the potential variability of using ICD-10 codes based on institutional/providers heterogeneity and differences. To overcome this, we included all cases of IVH and not only severe IVH and we ran separate analysis for each scenario to overcome variability in grading IVH techniques. In addition, the oversized sample size obtained through using the NIS datasets may overcome such variability by creating a national average to the condition examined. However, the study inherited some limitations. Information on timing, whether the PA was acute or chronic, was not available in the database. It is possible that the occurrence of total or partial PA histopathological can impact the condition of neonates or the outcome that follows, thus, an analysis to identify any differences in pathological findings based on these factors is needed in future studies. Another limitation is that we could not stratify the PA cases by severity as the non-stress test, Apgar score or umbilical cord blood gases were not available. Also, linkage to maternal record/codes and medications usage such as betamethasone and indomethacin were not available.

In conclusion, this study showed the association of placental abruption with increased prevalence of IVH and severe IVH in VLBW infants. Further studies are needed to identify whether prevention and early management of PA would ameliorate this association and its relationship with the neurodevelopmental outcome.

## Supplementary information

Supplementary Table 1

## Data Availability

The data that support the findings of this study are available from the National Inpatient Sample as part of the Healthcare Cost and Utilization Project. Restrictions apply to the availability of these data, which were used under license for this study. Data are available at https://www.hcup-us.ahrq.gov/.
