# Supplementary material for: Placental abruption and risk for intraventricular hemorrhage in very low birth weight infants: the United States national inpatient database
Source: J Perinatol. 2024 May 29;44(10):1504–8. doi: 10.1038/s41372-024-02017-y (PMC11442351; doi:10.1038/s41372-024-02017-y)
Supplement: Supplementary file 1 — Supplementary Table 1 [file 41372_2024_2017_MOESM1_ESM.docx]

**Supplemental Table (1):** ICD-10 codes used to identify the maternal and neonatal confounding variables in eth sample.

| **Maternal and neonatal factors** | **ICD-10 codes** |
| --- | --- |
| Maternal hypertension | P000 |
| Maternal diabetes mellitus | P700, P701 |
| Placenta Previa | P020 |
| Maternal chorioamnionitis | P027, P0270, P0278, P392, P398' |
| Breech presentation | P030 |
| Mal-presentation | P031 |
| Nuchal cord | P024 |
| Cord prolapse | P025 |
| Respiratory Distress Syndrome | P220, P229 |
| Apnea of Prematurity | P283, P284 |
| Pneumothorax | P251 |
| Pulmonary hemorrhage | P260, P261, P268, P269 |
| Pulmonary hypertension | P2930, P2938 |
| Anemia of prematurity | P612 |
| Necrotizing enterocolitis | P771, P772, P773, P779 |
| Sepsis | P360, P361, P3610, P3619, P362, P363, P3630, P3639, P364, P365, P368, P369, P372 |
